# The effect of environmental calcium on gene expression, biofilm formation and virulence of *Vibrio parahaemolyticus*

**DOI:** 10.3389/fmicb.2024.1340429

**Published:** 2024-05-31

**Authors:** Xue Li, Jingyang Chang, Miaomiao Zhang, Yining Zhou, Tingting Zhang, Yiquan Zhang, Renfei Lu

**Affiliations:** ^1^Department of Clinical Laboratory, Affiliated Nantong Hospital 3 of Nantong University, Nantong Third People’s Hospital, Nantong, China; ^2^School of Medicine, Jiangsu University, Zhenjiang, China

**Keywords:** *Vibrio parahaemolyticus*, calcium, biofilm, virulence, gene expression

## Abstract

Calcium (Ca^2+^) can regulate the swarming motility and virulence of *Vibrio parahaemolyticus* BB22. However, the effects of Ca^2+^ on the physiology of *V. parahaemolyticus* RIMD2210633, whose genomic composition is quite different with that of BB22, have not been investigated. In this study, the results of phenotypic assays showed that the biofilm formation, c-di-GMP production, swimming motility, zebrafish survival rate, cytoxicity against HeLa cells, and adherence activity to HeLa cells of *V. parahaemolyticus* RIMD2210633 were significantly enhanced by Ca^2+^. However, Ca^2+^ had no effect on the growth, swarming motility, capsular polysaccharide (CPS) phase variation and hemolytic activity. The RNA sequencing (RNA-seq) assay disclosed 459 significantly differentially expressed genes (DEGs) in response to Ca^2+^, including biofilm formation-associated genes and those encode virulence factors and putative regulators. DEGs involved in polar flagellum and T3SS1 were upregulated, whereas majority of those involved in regulatory functions and c-di-GMP metabolism were downregulated. The work helps us understand how Ca^2+^ affects the behavior and gene expression of *V. parahaemolyticus* RIMD2210633.

## Introduction

*Vibrio parahaemolyticus* (*V. parahaemolyticus*) inhabits naturally in the marine ecosystems and commonly causes seafood-associated gastroenteritis in human (Chen et al., 2022). It expresses different kinds of virulence factors, mainly including thermostable direct hemolysin (TDH), type III secretion system 1 (T3SS1), T3SS2, type VI secretion system 1 (T6SS1), T6SS2 and extracellular proteases (Cai and Zhang, 2018; Osei-Adjei et al., 2018; Li et al., 2019). TDH possesses the lethal toxicity, cytotoxicity, enterotoxicity, and hemolytic activity, and causes β-hemolysis on Wagatsuma agar, termed as the Kanagawa phenomenon (KP) (Cai and Zhang, 2018). T3SS1 possesses cytotoxicity and lethal activity, whereas T3SS2 is mainly involved in *V. parahaemolyticus*-induced enterotoxicity (Hiyoshi et al., 2010). T6SS1 has anti-bacterial activity and thus plays a role in environmental fitness of *V. parahaemolyticus*, whereas T6SS2 contributes to bacterial adhesion to host cells (Yu et al., 2012; Salomon et al., 2013). In addition, extracellular proteases may play roles in skin infections and processing other protein factors (Osei-Adjei et al., 2018). Other factors such as capsular polysaccharide (CPS), iron uptake system, and lipopolysaccharide are also involved in the virulence of *V. parahaemolyticus* (Li et al., 2019).

*V. parahaemolyticus* can form biofilms on the surface, which refer to bacterial communities that aid bacteria to survive in adverse conditions (Ruhal and Kataria, 2021; Sun et al., 2022). Flagellar systems are required for bacterial biofilm formation (Yildiz and Visick, 2009). *V. parahaemolyticus* can produce a single polar flagellum and numerous lateral flagella, which are designed for swimming in liquids and swarming over surfaces, respectively (McCarter, 2004). The polar flagellar gene mutant defects in biofilm formation with failure in the progress of three-dimensional expansion (Enos-Berlage et al., 2005). Type VI pili can promote bacteria to colonize on surfaces and thus are positively correlated with biofilm formation (Yildiz and Visick, 2009; Ruhal and Kataria, 2021). Mannose-sensitive haemagglutinin (MSHA) and chitin-regulated pili are type IV pili produced by *V. parahaemolyticus* that play roles in attachment and agglutination, respectively (Shime-Hattori et al., 2006). *V. parahaemolyticus* exopolysaccharide (EPS), which is biosynthesized by the products of *cpsA-K* and *scvA-O* (Liu et al., 2022), are the main components of biofilm matrix. The *cps* but not *scv* gene cluster is required for the wrinkly colony formation, as only the *cps* gene mutants form smooth colonies on the plate (Liu et al., 2022). *V. parahaemolyticus* undergoes the wrinkly and smooth phase variation, which affects biofilm formation and virulence factor production (Wu et al., 2022, 2023).

The second messenger cyclic diguanosine monophosphate (c-di-GMP) is widely used by bacteria to control gene expression, including those associated with biofilm formation and virulence (Mills et al., 2011). c-di-GMP is biosynthesized from guanosine triphosphate by the GGDEF domain of diguanylate cyclase (DGC) and degraded into linear di-GMP (pGpG) or GMP by the EAL or HD-GYP domain of phosphodiesterase (PDE) (Mills et al., 2011). In *V. parahaemolyticus*, there are 28 proteins with GGDEF domains, 13 proteins with EAL domains, 16 proteins with both GGDEF and EAL domains, and 5 proteins with HD-GYP domains (Romling et al., 2013). Therefore, a total of 62 proteins are inferred to be required for the metabolism of c-di-GMP in *V. parahaemolyticus*. However, only a few of them have been confirmed to regulate biofilm formation, motility and/or c-di-GMP metabolism, including the GGDEF-EAL-domain containing proteins ScrC and ScrG, GGDEF-domain containing proteins ScrO, ScrJ, ScrL, and GefA, and EAL-domain containing proteins LafV and TpdA (Boles and McCarter, 2002; Kim and McCarter, 2007; Kimbrough et al., 2020; Kimbrough and McCarter, 2021; Martinez-Mendez et al., 2021; Zhong et al., 2022).

It is necessary for bacteria to continuously exchange substances with the surroundings during growth, making them sensitive to changes in environmental factors. Calcium (Ca^2+^) is one of the most abundant metal ions in seawater and thus plays crucial roles in the survival, reproduction and behavior altering of marine microorganisms (Chodur et al., 2018). It was previously reported that Ca^2+^ strongly inhibited the biofilm formation of *V. cholerae* (Bilecen and Yildiz, 2009); however, it effectively increased EPS production and biofilm formation in *V. fischeri* and *V. vulnificus* (Garrison-Schilling et al., 2011; Tischler et al., 2018). In *V. parahaemolyticus* BB22, Ca^2+^ was also able to enhance the lateral flagellar gene expression and T3SS1 production, and thus heightened the swarming motility and the cytotoxicity toward host cells (Gode-Potratz et al., 2010). The lateral flagella-mediated swarming motility is required for the formation of 3D structural biofilm in vibrio species (Yildiz and Visick, 2009). Thus, Ca^2+^ may also have regulatory effects on biofilm formation in *V. parahaemolyticus*. In addition, the gene composition of *V. parahaemolyticus* RIMD2210633 is quite different with that of BB22, which harbors ~300 novel genes but lacks prophage f237 and several genomic islands (Jensen et al., 2013). Conflicting results on gene functions have also been observed in these two strains (Zhang et al., 2021a, 2023a). Therefore, roles of Ca^2+^ in gene expression and behavior altering are also worth investigating in *V. parahaemolyticus* RIMD2210633.

The aim of study was to explore the effects of Ca^2+^ on the behavior and gene expression of *V. parahaemolyticus* RIMD2210633. Notably enhancements in biofilm formation, intracellular c-di-GMP level, swimming motility, zebrafish survival rate, cytoxicity against HeLa cells and adhesion activity to HeLa cell monolayer were evident in response to Ca^2+^. However, no significant changes were observed for bacterial growth, swarming motility, CPS phase variation and hemolytic activity in response to Ca^2+^. In addition, Ca^2+^ significantly impacted on the expression of 459 genes in *V. parahaemolyticus* RIMD2210633, of these 265 genes were down-regulated and 194 genes were up-regulated. The work helps us understand how Ca^2+^ affects the behavior and gene expression of *V. parahaemolyticus* RIMD2210633.

## Materials and methods

### Bacterial strains and growth conditions

*V. parahaemolyticus* RIMD2210633 (Makino et al., 2003), termed here as the wild type (WT) strain, was used throughout in this study. *V. parahaemolyticus* strains were cultured in 2.5% (w/v) Bacto heart infusion (HI) broth (BD Biosciences, United States) at 37°C with shaking at 200 rpm. An overnight cell culture in HI broth was diluted 50-fold into 5 mL HI broth, followed by cultured at 37°C to OD_600_ = 1.4 (defined here as the second-round of culture). The resulting culture was diluted 1,000-fold into 5 mL HI broth or HI broth supplemented with 4 mM CaCl_2_ (HI-Ca^2+^) for the third round of growth (Gode-Potratz et al., 2010).

### Growth curve measurement

The growth curves of *V. parahaemolyticus* were measured similarly as previously described (Zhang et al., 2023b). Briefly, the second-round of culture was diluted 1,000-fold into 10 mL HI or HI-Ca^2+^ in glass tubes, and then grown continuously at 37°C with shaking at 200 rpm. The growth curves were created by measuring the OD_600_ values of each culture at 1 h intervals.

### Crystal violet staining for evaluating biofilm formation ability

Biofilm formation abilities of *V. parahaemolyticus* in HI and HI-Ca^2+^ broth were assessed by the crystal violent (CV) staining method, which was performed similarly as previously described (Zhang et al., 2023a). Briefly, the second-round of culture was diluted 50-fold into 2 mL HI broth or HI-Ca^2+^ in a 24-well cell culture plate, and then incubated at 30°C with shaking at 150 rpm for 6, 12, 24, 48, or 72 h. Culture solution containing the planktonic cells were collected for the measurement of OD_600_ values. Biofilm cells were washed gently with deionized water for three times, and then stained with 0.1% CV, followed by washed for another three times. Bound CV in each well was dissolved into 2.5 mL 20% acetic acid, and then the OD_570_ value was measured. Relative biofilm formation was expressed as OD_570_/OD_600_.

### Colony morphology assay

The colony morphology assays were performed similarly as previously described (Zhang et al., 2023a). Briefly, the second-round of culture was diluted 50-fold into 5 mL Difco marine (M) broth 2,216 (BD Biosciences, United States), and then incubated statically at 30°C for 48 h. Two microliter was taken to spot on an HI or HI-Ca^2+^ plate, and then incubated at 37°C for 24 h.

### Swimming motility

Swimming motility assays were performed similarly as previously described (Lu et al., 2021). Briefly, 2 μL of the second-round of culture were inoculated into a semi-solid HI plate containing 0.5% (w/v) Difco Noble agar (BD Biosciences, United States) or semi-solid HI plate supplemented with 4 mM CaCl_2_. The diameters of swimming areas were measured after incubation at 37°C.

### Swarming motility

Swarming motility assays were performed similarly as previously described (Lu et al., 2019). Briefly, 2 μL of the third-round of culture were spotted on a HI plate containing 2.0% (w/v) Difco noble agar or HI plate supplemented with 4 mM CaCl_2_. The diameters of swarming zones were measured after incubation at 37°C.

### Quantification of c-di-GMP

Intracellular c-di-GMP level was quantified similarly as previously described (Gao et al., 2020). Briefly, the second-round of culture was diluted 50-fold into 2 mL HI broth or HI-Ca^2+^ in a 24-well cell culture plate, and then incubated at 30°C with shaking at 150 rpm for 6, 12, 24, 48, or 72 h. Bacterial cells at each time point were simultaneously harvested in triplicate from planktonic fractions and biofilms, resuspended in 2 mL ice-cold phosphate buffered saline (PBS), boiled at 100°C for 5 min, and then sonicated for 15 min in an ice-water bath. The concentrations of c-di-GMP and total proteins in the supernatant was measured by using a c-di-GMP Enzyme-linked Immunosorbent Assay (ELISA) Kit (Mskbio, China) and a Pierce BCA Protein Assay kit (ThermoFisher Scientific, United States), respectively. Intracellular c-di-GMP levels were expressed as pmol/g protein.

### Detection of CPS phase variation

CPS phase variation was detected as previously study (Zhang et al., 2023a). Briefly, a small amount of overnight cell culture of *V. parahaemolyticus* RIMD2210633 was streaked onto a HI plate or HI plate supplemented with 4 mM Ca^2+^, and then statically incubated at 37°C for 24 h.

### Zebrafish infection assay

Zebrafish infection assays were performed similarly as previously described (Zhang et al., 2016). Briefly, *V. parahaemolyticus* was cultured in HI broth or HI-Ca^2+^ broth in a 24-well cell culture plate at 30°C with shaking at 150 rpm for 12 h. Bacterial cells were harvested, washed, resuspended in PBS, and then adjusted to 10^8^ CFU/mL. A total of 20 μL bacterial suspensions was intraperitoneally injected into each of the 15 wild-type AB adult zebrafishes (7–8 months). The number of dead zebrafishes was monitored at a 12 h interval. PBS was used as the negative control. The proposed animal experiments were approved by the Ethics Review Committee of Nantong University (approval number: P20230206-004).

### Cytotoxicity against HeLa cells

Cytotoxicity assay was performed similarly as previously described (Zhang et al., 2018). Briefly, the second-round of culture was diluted 50-fold into 2 mL HI broth or HI-Ca^2+^ broth in a 24-well cell culture plate, and then incubated at 30°C with shaking at 150 rpm for 12 h. Bacterial cells were harvested, washed, and then diluted serially with the pre-warmed Dulbecco’s modified Eagle’s medium (DMEM) lacking phenol red for CFU determination and infection. HeLa cells, which are preserved in our lab, were infected with 10^6^ CFU of bacterial cells for 3 h at a multiplicity of infection (MOI) of 2.5 (bacterial cells: HeLa cells). The released lactate dehydrogenase (LDH) was quantified with a CytoTox 96^®^ Non-Radioactive Cytotoxicity Assay kit (Promega, United States) according to the manufacturer’s instructions.

### Adhesion against HeLa cells

HeLa cell adhesion assays were performed similarly as previously described (Zhang et al., 2017a). Briefly, the second-round of culture was diluted 50-fold into 2 mL HI broth or HI-Ca^2+^ broth in a 24-well cell culture plate, and then incubated at 30°C with shaking at 150 rpm for 12 h. Bacterial cells were harvested and re-suspended in DMEM. HeLa cell monolayers were maintained in DMEM containing 10% fetal bovine serum (FBS, Invitrogen) at 37°C with 5% CO_2_. The cell monolayers were infected at a MOI of 10. After incubation for 90 min, the monolayers were washed three times with PBS, and then lysed with 1% Triton X-100. The number of bacterial cells in the lysates were serially diluted and counted on LB agar plates. The CFU of input bacterial cells were also determined by the plate count method. Percent adherence was calculated as adhered bacterial cells/input bacterial cells.

### The Kanagawa phenomenon test

KP tests were performed similarly as previously described (Sun et al., 2022). Briefly, 5 μL of the second-round of bacterial culture were inoculated onto a Wagatsuma agar (CHROMagar, China) containing 5% rabbit red blood cells (RBCs) or 5% RBCs together with 4 mM Ca^2+^. The radius of each β-hemolysin zone was determined after incubation at 37°C for 24 h.

### RNA extraction and RNA sequencing

The second round of culture was diluted 50-fold into 2 mL HI broth and HI-Ca^2+^, respectively, in a 24-well cell culture plate, and then incubated at 30°C with shaking at 150 rpm for 12 h. Bacterial cells were simultaneously harvested in triplicate from planktonic fractions and biofilms for total RNA preparation using TRIzol Reagent (Invitrogen, United States). RNA concentration was determined by a Nanodrop 2000, and RNA integrity was measured by agarose gel electrophoresis. rRNA was removed and mRNA was enriched by using an Illumina/Ribo-Zero^™^ rRNA Removal Kit (bacteria) (Illumina, United States). cDNA library was constructed and sequenced on an Illumina Hiseq platform in GENEWIZ Biotechnology Co. Ltd. (Suzhou, China) (Wu et al., 2022). Raw data of RNA-seq was filtered by Cutadapt (v1.9.1), and then aligned with the genome of *V. parahaemolyticus* RIMD2210633 using Bowtie2 (v2.2.6) (Bray et al., 2016). Gene expression in bacterial cells grown in HI-Ca^2+^ (test group) was compared with that in bacterial cells grown in HI broth (reference group). HTSeq (v0.6.1) and Fragments Per Kilo bases per Million reads (FPKM) were used to calculate gene expression (Mortazavi et al., 2008; Anders et al., 2015). DESeq (v1.6.3) was used to analyze the difference in gene expression with selection criteria of qvalue (fdr, padj) ≤ 0.05 and absolute fold change ≥ 2 (Love et al., 2014). Gene Ontology (GO) functional annotation was performed to analyze the differentially expressed genes (DEGs) involved in cellular components, molecular functions and biological processes (Harris et al., 2004). Kyoto Encyclopedia of Genes and Genomes (KEGG) pathway enrichment was performed to analyze DEGs involved in metabolic pathways (Kanehisa and Goto, 2000). The putative functions of proteins that are encoded by DEGs were predicted by the Cluster of Orthologous Groups of proteins (COG) database (Tatusov et al., 2000).

### Statistical methods

Each phenotypic assay was performed at least three times with three biological replicates in each. qPCR and c-di-GMP quantification were performed three times, respectively, and the results were expressed as the mean ± standard deviation (SD). Paired Student’s *t-*test was applied to calculate statistical significance, and *p <* 0.05 was considered as significant.

## Results

### Ca^2+^ did not affect the growth of *Vibrio parahaemolyticus*

**Figure 1:**
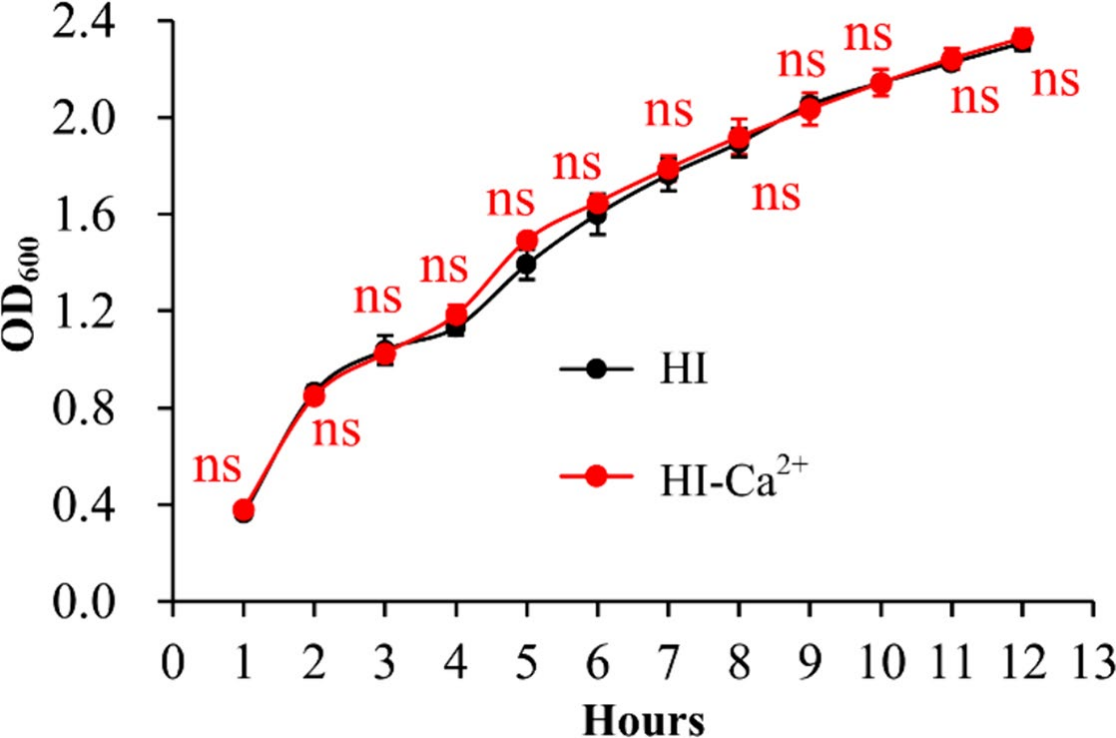
Growth curves of *V. parahaemolyticus*. The OD_600_ values of *V. parahaemolyticus* RIMD2210633 grown with shaking in HI broth or HI broth supplemented with 4 mM CaCl_2_ were monitored at 1 h intervals. Experiments were performed three times with three replicates per trial for each condition. Paired Student’s *t-*tests were used to calculate statistical significance. The “ns” means no statistically significant differences (*p* > 0.05).

The growth curves of *V. parahaemolyticus* in HI broth and HI-Ca^2+^ was measured to assess whether Ca^2+^ affects the bacterial growth. As shown in Figure 1, the growth rate of *V. parahaemolyticus* in HI-Ca^2+^ has no significant difference with that in HI broth, suggesting that additional Ca^2+^ did not influence the growth of *V. parahaemolyticus*.

### Ca^2+^ promotes biofilm formation by *Vibrio parahaemolyticus*

**Figure 2:**
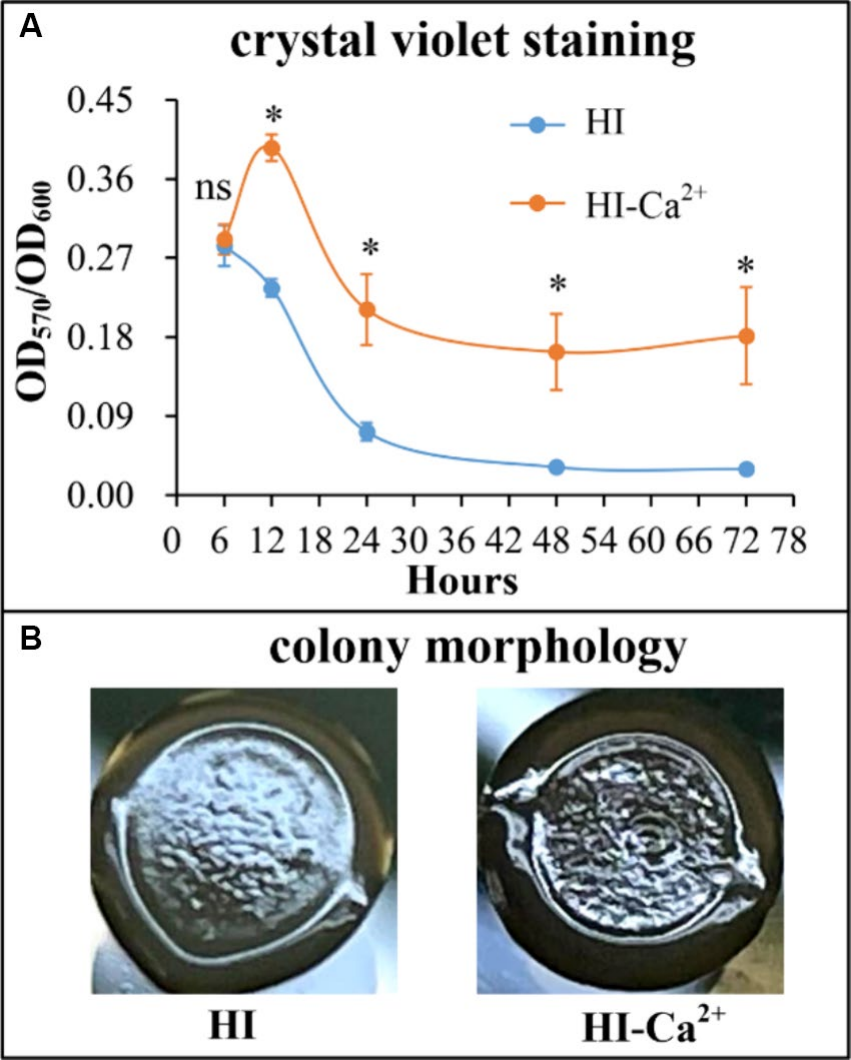
Involvement of Ca^2+^ in biofilm formation by *V. parahaemolyticus* RIMD2210633. Crystal violet staining **(A)** and colony morphology **(B)** of *V. parahaemolyticus* were determined. Pictures are representative of three independent experiments with three replicates each. Paired Student’s *t-*tests were used to calculate statistical significance. The asterisks (*) indicate statistically significant differences (*p* < 0.05), whereas the “ns” represents no statistically significant differences (*p* > 0.05).

The crystal violent (CV) staining and colony morphology assays were performed to investigate whether environmental Ca^2+^ affects biofilm formation by *V. parahaemolyticus*. As shown in Figure 2A, *V. parahaemolyticus* was able to form biofilms in both HI and HI-Ca^2+^ broth; however, the biofilm amounts in HI broth declined continuously with the incubation time, while those in HI-Ca^2+^ broth first increased considerably and then decreased dramatically, and the highest amount of biofilm occurred at the 12th h; the bacterial cells grown in HI-Ca^2+^ produced significantly more biofilms relative to those grown in HI broth (*p* < 0.05) at all time points tested except for the 6th h. As further determined by colony morphology assay, the colonies of *V. parahaemolyticus* grown on the HI-Ca^2+^ plate more wrinkled than those on the HI plate (Figure 2B). These results suggested that additional Ca^2+^ is beneficial for biofilm formation by *V. parahaemolyticus*.

### Ca^2+^ increases swimming motility of *Vibrio parahaemolyticus*

**Figure 3:**
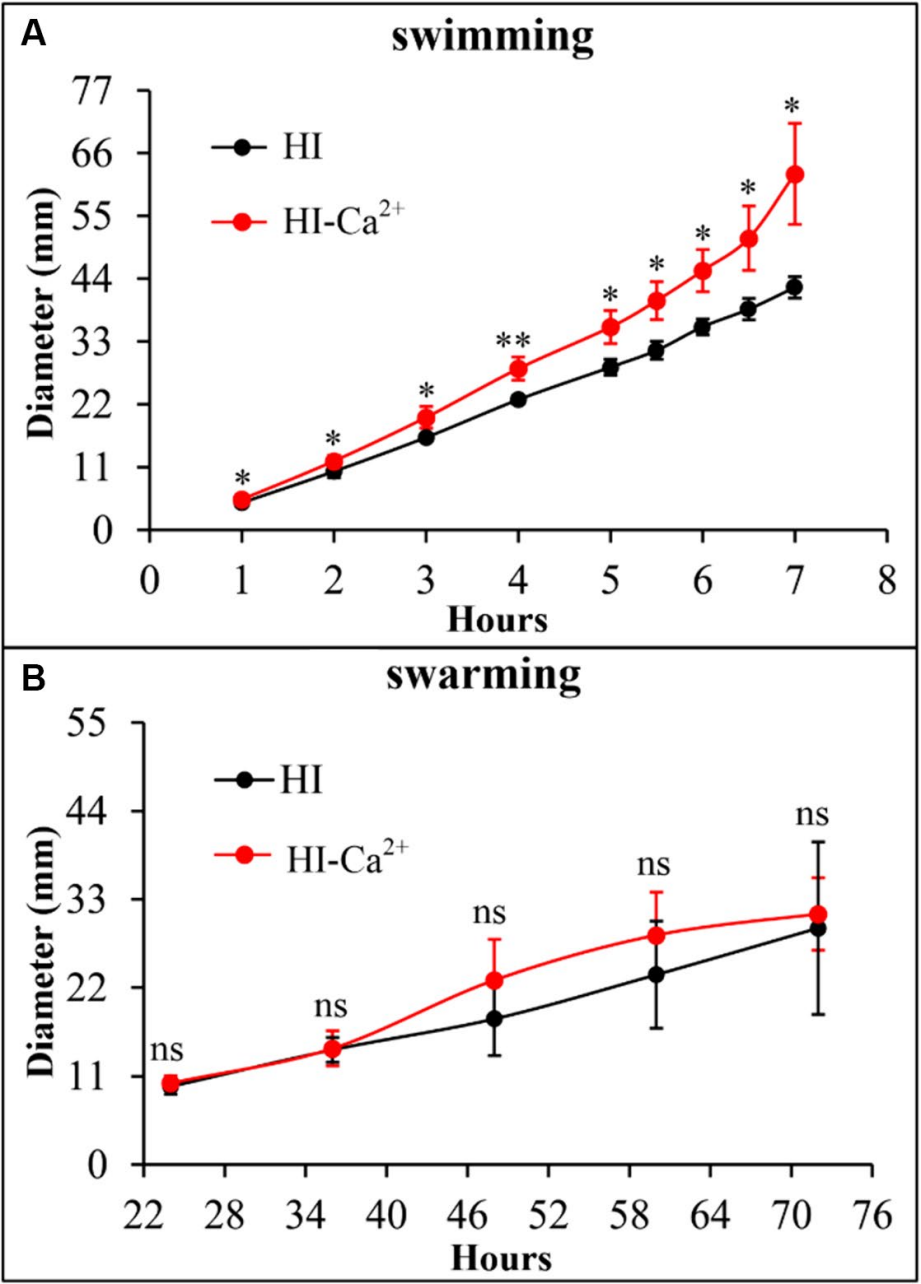
Ca^2+^ promoted the swimming motility of *V. parahaemolyticus* RIMD2210633. Swimming **(A)** or swarming **(B)** capacity of *V. parahaemolyticus* was measured by detection of the diameters of swimming or swarming areas in a semi-solid swimming or on swarming agar. The data at each time point are expressed as the mean ± SD of three independent experiments with three replicates each. Paired Student’s *t-*tests were used to calculate statistical significance. The single asterisk (*) represents *p* < 0.05, whereas the “ns” indicates *p* > 0.05.

The swimming and swarming motility were investigated to assess whether Ca^2+^ affects the motor capacities of *V. parahaemolyticus*. As shown in Figure 3A, swimming motility was significantly enhanced in the condition of HI-Ca^2+^ compared with that of HI at all time points tested. However, there was no significant differences in swarming motility between the two growth conditions at all time points tested (Figure 3B). These results suggested that Ca^2+^ enhanced the swimming capacity of *V. parahaemolyticus*, but had no effect on swarming motility.

### Ca^2+^ increases intracellular c-di-GMP level of *Vibrio parahaemolyticus*

**Figure 4:**
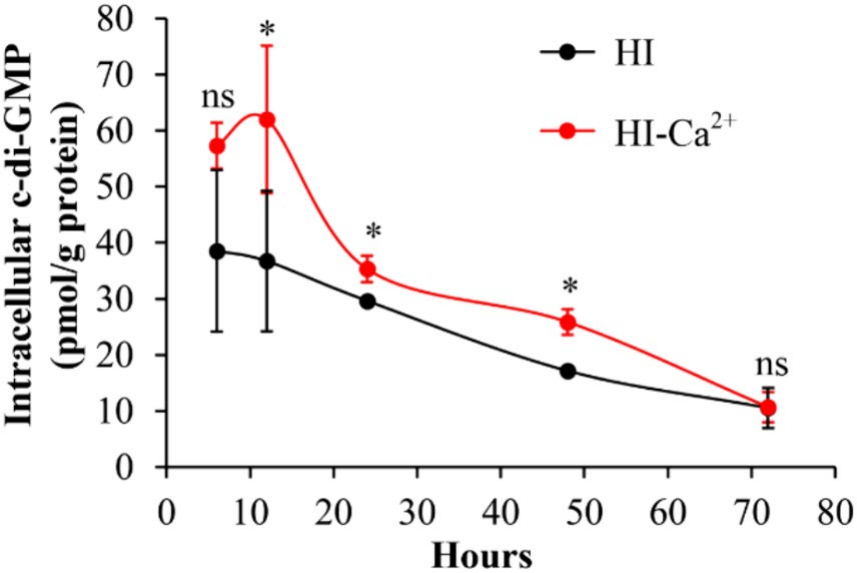
Ca^2+^ enhanced the production of intracellular c-di-GMP in *V. parahaemolyticus* RIMD2210633. Bacterial cells were cultured 30°C with shaking at 150 rpm in HI broth containing 0 or 4 mM CaCl_2_, and then harvested simultaneously in triplicate from biofilms and planktonic fractions. The intracellular c-di-GMP was extracted by ultrasonication, and the c-di-GMP concentration was measured by a c-di-GMP Enzyme-linked Immunosorbent Assay (ELISA) Kit. Intracellular c-di-GMP level was expressed as pmol/mg. The data are expressed as the mean ± SD of three independent experiments with three replicates each. Paired Student’s *t-*tests were used to calculate statistical significance. The single asterisk (*) represents *p* < 0.05, whereas the “ns” indicates *p* > 0.05.

c-di-GMP is involved in the regulation of multiple bacterial behaviors, including biofilm formation (Biswas et al., 2020). Therefore, we measured intracellular c-di-GMP levels to investigate whether Ca^2+^-enhanced biofilm formation is related to the variation of c-di-GMP level. As shown in Figure 4, the intracellular c-di-GMP levels were significantly increased in bacterial cells grown in HI-Ca^2+^ compared to those grown in HI broth at the incubation time from 12 to 48 h. However, there were no significant differences in c-di-GMP levels in *V. parahaemolyticus* grown under the two conditions at the 6th and 72nd h. These results suggested that Ca^2+^ enhanced the biosynthesis of c-di-GMP in *V. parahaemolyticus*.

### Ca^2+^ did not affect CPS phase variation of *Vibrio parahaemolyticus*

**Figure 5:**
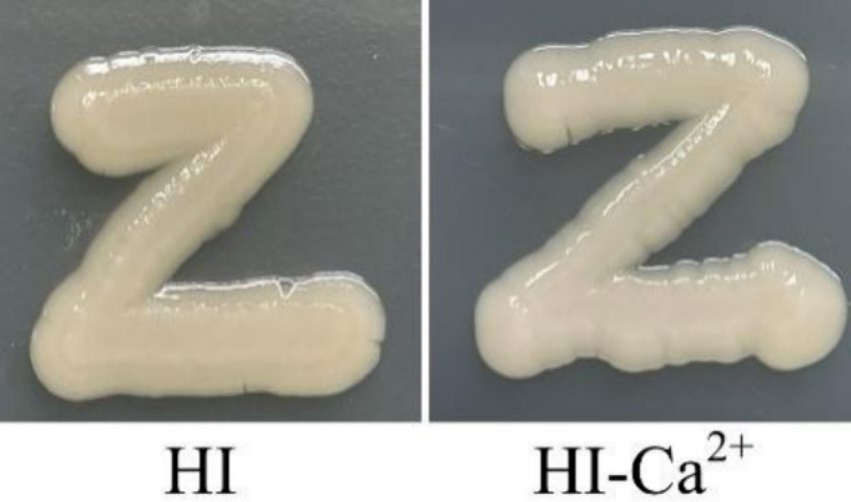
Ca^2+^ did not affect the CPS phase variation of *V. parahaemolyticus* RIMD2210633. Bacterial cells were streaked onto a HI plate containing 0 or 4 mM CaCl_2_, and then statically incubated at 37°C for 24 h. Pictures were representative of two independent experiments with three replicates each.

The switching between opaque (OP) and translucent (TR) colony phenotype of *V. parahaemolyticus* is directly attributed to the production of CPS or not and thus was termed as the CPS phase variation (Enos-Berlage and McCarter, 2000). CPS affects biofilm formation and virulence of *V. parahaemolyticus* (Hsieh et al., 2003; Lee et al., 2013). Therefore, CPS phase variation was assessed to detect whether Ca^2+^ affects CPS production in *V. parahaemolyticus*. As shown in Figure 5, *V. parahaemolyticus* grown on both HI and HI-Ca^2+^ agars exhibited OP phenotype, suggesting that Ca^2+^ did not affect CPS phase variation and CPS production in *V. parahaemolyticus*.

### Ca^2+^ affects the virulence of *Vibrio parahaemolyticus*

**Figure 6:**
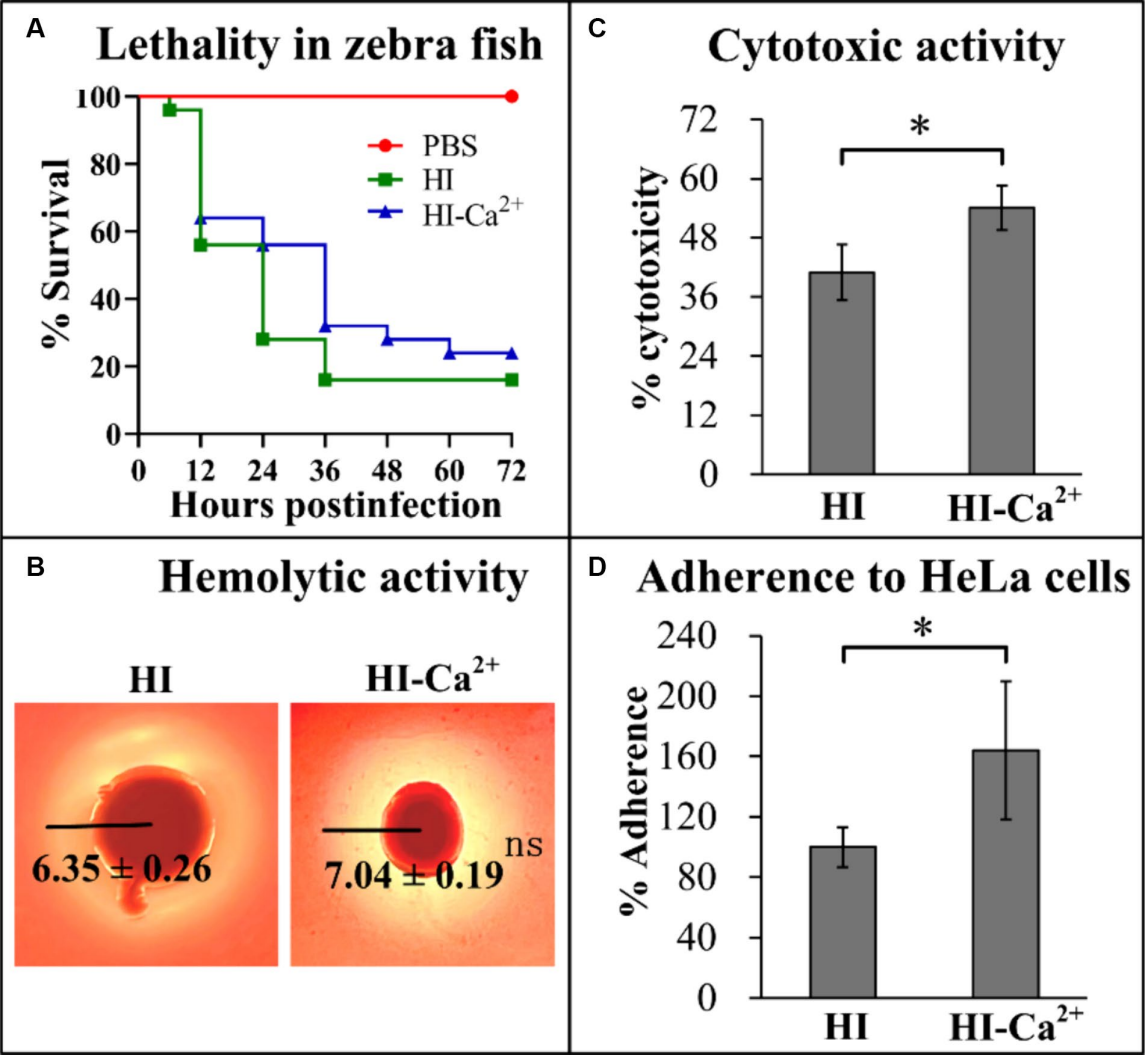
Regulatory effects of Ca^2+^ on the virulence of *V. parahaemolyticus* RIMD2210633. The numeral results were expressed as the mean ± SD from at least two independent experiments with four replicates. Paired Student’s *t-*tests were used to calculate statistical significance. The single asterisk (*) represents *p* < 0.05, whereas the “ns” indicates *p* > 0.05. **(A)** Survival curves of zebrafish. Approximately 2 × 10^6^ CFU *V. parahaemolyticus* cells were injected intraperitoneally into each adult zebrafish, and then the survival rates of zebrafishes were recorded with a 12 h interval. **(B)** Hemolytic activity. Effects of Ca^2+^ on the hemolytic activity of *V. parahaemolyticus* RIMD2210633 was assessed by KP test. The pictures shown here are representative images of three independent experiments with four replicates in each. **(C)** Cytotoxicity against HeLa cells. The cytotoxicity of *V. parahaemolyticus* RIMD2210633 against HeLa cells was evaluated in terms the release of LDH. **(D)** Adherence against HeLa cells. HeLa cells were infected with *V. parahaemolyticus* RIMD2210633 at a MOI of 10. The percent adherence was calculated as bacterial cells adhered/input bacterial cells. The adherence rate of bacterial cells cultured in HI broth was normalized to 100%.

Several virulence-associated phenotype assays were performed to elucidate the potential effects of Ca^2+^ on the virulence of *V. parahaemolyticus*. The final survival rate of zebrafishes that were infected by *V. parahaemolyticus* grown in HI broth was 16%, in comparison to the survival rate of 24% for those were infected by *V. parahaemolyticus* grown in HI-Ca^2+^, while a 100% survival rate was observed for the control group (Figure 6A). In addition, it is worth noting that the survival rates were continuous gradient decreasing until stabilization in both HI and HI-Ca^2+^ groups with the passage of post-infection time, especially in the HI-Ca^2+^ group (Figure 6A). The results of KP test showed that the diameters of β-hemolysis zones caused by *V. parahaemolyticus* grown on the Ca^2+^-containing condition were similar to those of on the condition without Ca^2+^, suggesting that Ca^2+^ had no regulatory effect on the hemolytic activity of *V. parahaemolyticus* (Figure 6B). In addition, the cytotoxicity and cell adhesion ability of *V. parahaemolyticus* grown in HI-Ca^2+^ broth were all significantly enhanced relative to those grown in HI broth, suggesting that Ca^2+^ was able to induce the cytotoxicity and cell adhesion ability of *V. parahaemolyticus* against HeLa cells (Figures 6C,D). Taken together, Ca^2+^ was able to affect the virulence of *V. parahaemolyticus.*

### Overview of *Vibrio parahaemolyticus* gene expression in the response to Ca^2+^

**Figure 7:**
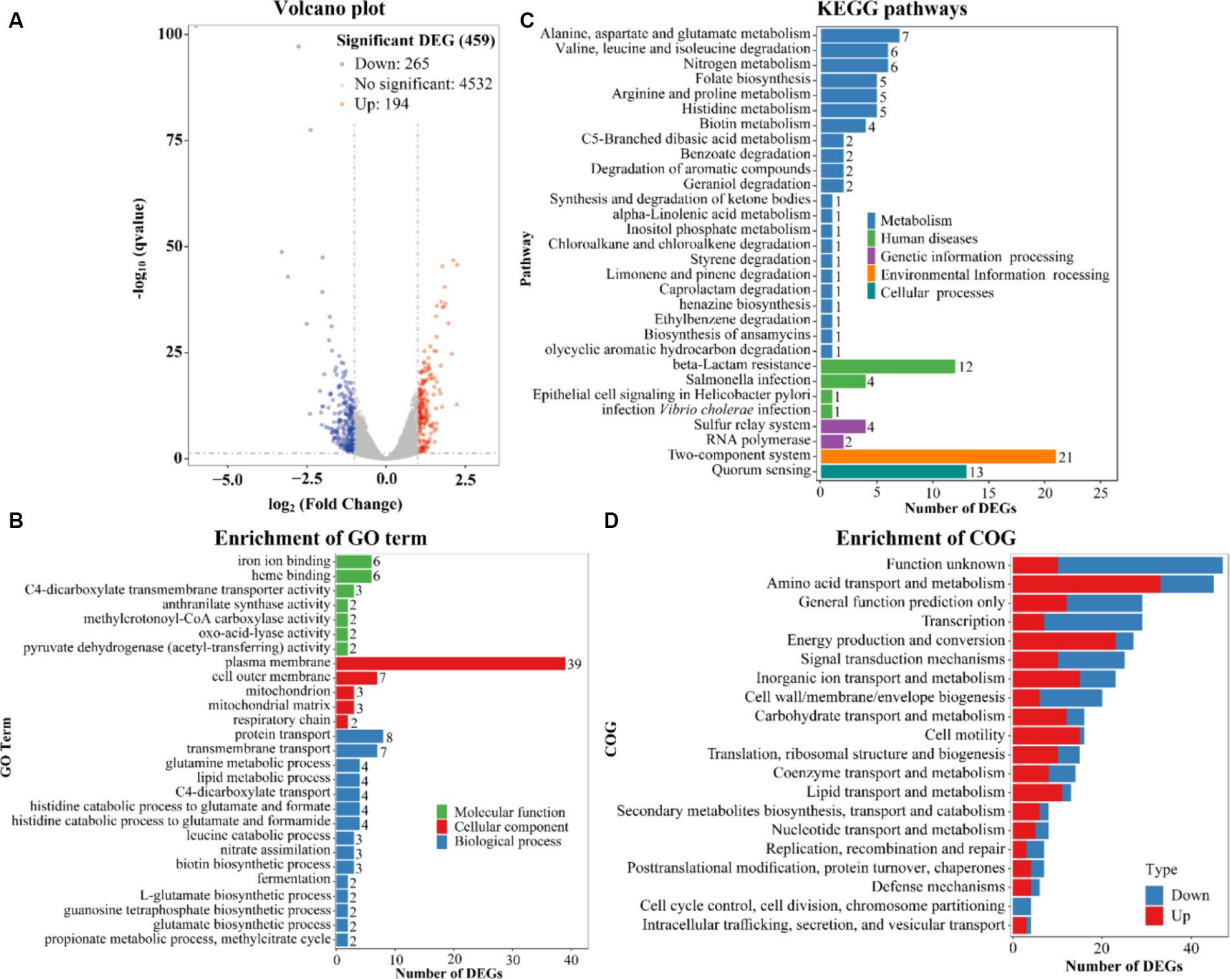
Expression profiles of *V. parahaemolyticus* RIMD2210633 in the response of Ca^2+^. **(A)** Volcano plot. Red, blue and gray points represent the up-regulated, down-regulated and no-significant regulated genes, respectively. **(B)** The enrichment of gene ontology (GO) term. Green, red and blue bars represent molecular function, cellular component and biological process, respectively. The number on the top of each bar indicates the number of enriched genes. **(C)** Pathways of differentially expressed genes were enriched by Kyoto Encyclopedia of Genes and Genomes (KEGG). The vertical axis represents KEGG classification, while the horizontal axis represents the number of DEGs. **(D)** Cluster of Orthologous Groups of proteins (COG). The vertical axis represents COG classification, whereas the horizontal axis represents the number of DEGs. Red and blue bars represent up-regulated and down-regulated genes, respectively.

The highest biofilm amount of *V. parahaemolyticus* in HI-Ca^2+^ occurred at the 12th h post-incubation (Figure 1A). Therefore, the gene expression profile of *V. parahaemolyticus* grown in HI-Ca^2+^ (test) for 12 h was compared with that grown in HI broth (reference) by RNA-seq to investigate the Ca^2+^ stimulon. The expression levels of 459 genes were differentially expressed in response to Ca^2+^. Of these, 194 were upregulated, and 265 were downregulated (Figure 7A). The results of GO enrichment showed that DEGs were enriched in biological process (15 GO terms, 54 DEGs), cellular component (5 GO terms, 54 DEGs) and molecular function (7 GO terms, 23 DEGs) (Figure 7B). The KEGG enrichment demonstrated that there are 57 DEGs involved in metabolism, 18 DEGs in human disease, 6 DEGs in genetic information processing, 21 DEGs in environmental information processing, and 13 DEGs in cellular processes (Figure 7C). The COG enrichment divided DEGs into at least 20 functional categories including function unknown, general function prediction only, amino acid transport and metabolism, transcription, and energy production and conversion (Figure 7D). The fold change, *p* value and descriptions of DEGs are listed in Supplementary Table S1.

### Selected DEGs from the Ca^2+^ stimulon

A total of 27 genes encoding putative regulators were remarkably differentially expressed in response of Ca^2+^ stimulation (Table 1). Of these, 24 were downregulated, and 3 were upregulated. Six DEGs encoding GGDEF- or EAL-domain containing proteins were remarkably differentially expressed, of these 1 was upregulated (VP1478) and 5 were downregulated (VP1881, VP2888, VPA0360, VPA0713, and VPA0925). In addition, 1 MSHA gene, 12 polar flagellar genes, and 9 T3SS1 genes were significantly upregulated in response of Ca^2+^ stimulation (Table 1). Moreover, 2 T3SS2 genes and 1 T6SS1 gene were downregulated in the response of Ca^2+^ stimulation (Table 1).

**Table 1:** Selected DEGs.

Gene ID	Name	Fold change	Functional annotation/domains
**Putative regulators**			
VP0118		3.9082	Nitrogen regulation protein
VP0350	*calR*	0.3036	Leucine transcriptional activator
VP0569		0.4666	DNA-binding response regulator PhoB
VP0709	*treR*	0.4999	Trehalose repressor
VP0938		0.4976	Transcriptional regulator
VP1190		3.2011	Anaerobic nitric oxide reductase regulator
VP1212		0.3269	DNA-binding response regulator
VP1889		0.3891	Cold shock transcriptional regulator CspA
VP2424		0.4934	AraC family transcriptional regulator
VP2450		0.3638	MarR family transcriptional regulator
VP2766		0.3554	Transcriptional repressor protein MetJ
VP2836		0.4025	TetR family transcriptional regulator
VPA0369		0.4607	LuxR family transcriptional regulator
VPA0462		0.4194	Predicted transcriptional regulators
VPA0495		2.1037	AraC family transcriptional regulator
VPA0531		0.4994	AraC family transcriptional regulator
VPA0599		0.4619	LysR family transcriptional regulator
VPA0601		0.4630	Arylsulfatase regulator
VPA0678		0.4243	Winged helix-turn-helix domain-containing protein
VPA0733		0.4453	LysR family transcriptional regulator
VPA0741		0.4154	Transcriptional regulator
VPA0743		0.4468	Response regulator VieB
VPA0785		0.4774	Transcriptional regulator
VPA1332	*vtrA*	0.4586	Transcriptional regulator ToxR
VPA1563		0.4925	Transcriptional regulator
VPA1682		0.4791	MarR family transcriptional regulator
VPA1729		0.3677	LuxR family transcriptional regulator
**c-di-GMP metabolism**			
VP1881		0.3659	EAL-only
VP2888		0.4291	GGDEF-only
VPA0360		0.3528	GGDEF-only
VPA0713		0.4602	EAL-only
VPA0925		0.4899	GGDEF-only
VPA1478		2.2069	GGDEF-only
**Type IV pili**			
VP2705	*mshK*	2.1165	MSHA biogenesis protein MshK
**T3SS1**			
VP1667	*vopN*	2.3288	Outer membrane protein PopN
VP1669	*vscO*	2.0976	Type III secretion protein YscO
VP1670	*vscP*	2.2189	Translocation protein in type III secretion
VP1671	*vscQ*	2.4952	Type III secretion system protein
VP1686	*vopS*	2.7630	Adenosine monophosphate-protein transferase
VP1695	*vscD*	2.2001	Type III export protein PscD
VP1696	*vscC*	2.7988	Type III secretion protein YscC
VP1697	*vscB*	2.2416	Type III export apparatus protein NosA
VP1698	*exsD*	2.0652	Hypothetical protein
**T3SS2**			
VPA1332	*vtrA*	0.4586	Transcriptional regulator ToxR
VPA1333		0.3990	Hypothetical protein
**T6SS1**			
VP1409		0.4972	Hypothetical protein
**Cell motility**			
VP0777	*flgD*	2.2977	Flagellar basal body rod modification protein
VP0778	*flgE*	2.3423	Flagellar hook protein FlgE
VP0781	*flgG*	3.5421	Flagellar basal body rod protein FlgG
VP0782	*flgH*	3.2884	Flagellar basal body L-ring protein
VP0786	*flgL*	2.0792	Flagellar hook-associated protein FlgL
VP0788	*flaC*	2.1250	Flagellin
VP2229	*cheA*	2.0450	Chemotaxis protein CheA
VP2248	*fliG*	2.1219	Flagellar motor switch protein G
VP2251	*flaM*	2.1576	FlaM
VP2257	*flaG*	2.5005	Flagellar protein FlaG
VP2258	*flaA*	2.1478	Flagellin
VPA1535	*fliG*	2.2787	Flagellar motor switch protein G

## Discussion

*V. parahaemolyticus* is ubiquitous in marine ecosystems, while Ca^2+^ is one of the most abundant metal ions in seawater (Chodur et al., 2018). Therefore, the fluctuation of Ca^2+^ concentration should be pertiment to the lifestyle, population density, and pathogenicity of *V. parahaemolyticus*. Indeed, Ca^2+^ is an important stimulus signal for *V. parahaemolyticus*. A study showed that Ca^2+^ affected the transcriptome and promoted swarming motility and T3SS1-dependent virulence of *V. parahaemolyticus* BB22 (Gode-Potratz et al., 2010). However, another study demonstrated that extracellular Ca^2+^ inhibited the expression of T3SS1 gene in *V. parahaemolyticus* RIMD2210633 (Sarty et al., 2012; Liu and Thomas, 2015). Thus, Ca^2+^-dependent gene expression in *V. parahaemolyticus* should be affected by the genetic background. In this study, we showed that addition of Ca^2+^ remarkably induces the c-di-GMP production, biofilm formation, swimming motility, zebrafish survival rate, cytoxicity and adhesion activity of *V. parahaemolyticus* RIMD2210633 (Figures 1, 3, 4, 6). However, Ca^2+^ has no regulatory effects on bacterial growth, swarming motility, CPS production and hemolytic activity (Figures 2, 4, 6). The data of RNA-seq showed that addition of Ca^2+^ strikingly influenced the expression of 459 genes in *V. parahaemolyticus* RIMD2210633. Of these, 265 were down-regulated and 194 were up-regulated (Figure 7; Supplementary Table S1). A previous study probed the response of *V. parahaemolyticus* BB22 to Ca^2+^ during growth on the surface and showed that only 50 genes were differentially expressed in response to Ca^2+^, including 35 up-regulated and 15 down-regulated genes (Gode-Potratz et al., 2010). The significant difference in the number of DEGs between the two works may be due to the following reasons: firstly, differences in the genomes of two strains (Jensen et al., 2013); secondly, gene expression profiles vary under different growth conditions; thirdly, the detection efficiency of RNA-seq used in this study for gene expression may be higher than that of microarray analysis used in the previous work.

Environmental Ca^2+^-dependent biofilm formation has been reported in vibrio species. Addition of Ca^2+^ led to *V. cholerae* to form a biofilm with decreased thickness and stability but increased roughness, and to express less VPS biosynthesis-associated proteins (Bilecen and Yildiz, 2009). Ca^2+^ is a potent inhibitor of VieA in *V. cholerae*, which is a EAL-domain containing protein that presumably decreases the c-di-GMP level (Tamayo et al., 2005). In *V. vulnificus*, Ca^2+^ remarkably enhances the rate of CPS and EPS phase variation, intracellular c-di-GMP concentrations, *brp* expression and biofilm formation (Garrison-Schilling et al., 2011; Chodur et al., 2018). IamR promotes the biofilm formation of *V. vulnificus* via activation of *iamA* expression, in a Ca^2+^-dependent manner (Pu et al., 2020). The Ca^2+^-binding protein CabA also contributes to the biofilm formation of *V. vulnificus* in a Ca^2+^-dependent manner under the conditions with elevated c-di-GMP levels (Park et al., 2015). Ca^2+^ is also able to induce *V. vulnificus* to form biofilms under non-conditions that typically do not form biofilms (Tischler et al., 2018). Herein, the data showed that Ca^2+^ induces the biofilm formation and wrinkly colony phenotype formation of *V. parahaemolyticus* RIMD2210633 (Figure 1). *V. parahaemolyticus* undergoes the wrinkly and smooth colony phase variation, which is directly associated with *cpsA-K*, and the wrinkly phenotype strain has a stronger biofilm formation ability than the smooth phenotype strain (Wu et al., 2022). However, Ca^2+^ seems to have no regulatory effect on *cpsA-K* transcription. Instead, 6 genes encoding EAL- or GGDEF-domain containing proteins were remarkably differentially expressed in response of Ca^2+^. Of these, 5 were downregulated (VP1881, VP2888, VPA0360, VPA0713, and VPA0925) and 1 was upregulated (VPA1478). VP1881, which was named as TpdA, is an EAL-domain containing protein that positively controls swimming and swarming motility, and negatively controls biofilm formation (Martinez-Mendez et al., 2021). VPA0360 (*scrM*) encoding a GGDEF-domain containing protein is part of s*crMNO* has been identified as a significant contributor to *V. parahaemolyticus* biofilm formation (Kimbrough et al., 2020). The functions of the other 4 genes are not yet known, but it can be speculated that one of the mechanisms by which Ca^2+^ promotes biofilm formation is to alter the intracellular c-di-GMP pool, as addition of Ca^2+^ to the medium induced the production of c-di-GMP (Figure 3). Additionally, 1 type IV pili gene (VP2705), 1 lateral flagellar gene (VPA1535) and 11 polar flagellar genes, which are all associated with biofilm formation (Yildiz and Visick, 2009), were remarkably induced by environmental Ca^2+^ (Table 1). There are 16 MSHA genes, 39 lateral flagellar genes, and 58 polar flagellar genes in the genome of *V. parahaemolyticus* RIMD2210633 (Makino et al., 2003). Thus, Ca^2+^ is unlikely to affect the assembly of type IV pili and lateral flagella as it only regulates one of the associated genes. However, Ca^2+^ should be able to affect the function of polar flagellum as it can induce swimming motility of *V. parahaemolyticus* (Figure 3A). In brief, Ca^2+^-dependent biofilm formation may be mediated by controlling c-di-GMP production and polar flagellum assembly in the current growth conditions. However, it must be noted that transcriptome data only explores gene expression profile at a certain growth phase and cannot fully reflect that during biofilm formation induced by Ca^2+^.

Previously, a study showed that Ca^2+^ induces bile salt-dependent virulence gene expression of *V. cholerae* through promoting bile salt-induced TcpP-TcpP interaction (Hay et al., 2017). Another study in *V. fischeri* showed that Ca^2+^ functions as a pH-dependent cue to promote the T6SS-mediated competition in low-viscosity liquid environments (Speare et al., 2022). The data presented here showed that Ca^2+^ can also regulate the virulence-associated phenotypes of *V. parahaemolyticus* RIMD2210633, including lethality in zebrafish, cytoxicity against HeLa cells and adhesion toward HeLa cells (Figure 6). However, only 12 genes encoding the known virulence determinants were remarkably differentially expressed in response to Ca^2+^, including 9 T3SS1 genes, 2 T3SS2 genes and 1 T6SS1 gene (Table 1). T3SS1 contributes cytotoxicity and lethal activity of *V. parahaemolyticus*, whereas T3SS2 is required for enterotoxicity (Hiyoshi et al., 2010). In addition, T6SS1 mainly contributes to the environmental fitness of *V. parahaemolyticus* as it possesses the anti-bacterial activity (Salomon et al., 2013). However, T3SS1, T3SS2, and T6SS1 are all multi gene encoded secretion systems, with each gene loci containing dozens of coding genes (Makino et al., 2003). Controlling several genes within the loci may not effectively affect the function of the secretion systems. Although Ca^2+^ was able to affect the virulence-related phenotypes (Figure 6), the growth conditions for phenotype experiments were not completely consistent with that of RNA-seq. In addition, a previous study has shown that Ca^2+^ activates T3SS1 gene expression in *V. parahaemolyticus* RIMD2210633 (Sarty et al., 2012; Liu and Thomas, 2015), while this study shows inhibitory effects, which may be due to the different growth conditions in these two works. The regulatory mechanisms of Ca^2+^ on the virulence of *V. parahaemolyticus* RIMD2210633 still needs to be investigated.

RNA-seq data also showed that 27 putative regulatory genes were remarkably differentially expressed in response to Ca^2+^, including 24 down-regulated and 3 up-regulated genes (Table 1). Some of these genes encode global regulators, including LysR family transcriptional regulators (VPA0599 and VPA0733), AraC family transcriptional regulators (VP2424, VPA0495, and VPA0531), LuxR family transcriptional regulators (VPA0369 and VPA1729) and MarR family transcriptional regulators (VP2450 and VPA1682). The two well-studied regulatory genes, *calR* and *vtrA*, were also significantly inhibited by Ca^2+^. CalR was originally identified in *V. parahaemolyticus* BB22 that positively regulated swarming motility and T3SS1 expression in the low-Ca^2+^ growth condition (Gode-Potratz et al., 2010). Later studies in *V. parahaemolyticus* RIMD2210633 revealed that it is a global regulator controlling multiple gene loci, including virulence genes and biofilm formation-associated genes (Osei-Adjei et al., 2017; Zhang et al., 2017a,b, 2021b). VtrA and VtrC form a co-component signal transduction system sensing the bile acid signals to positively regulates the expression of TDH and T3SS2 (Gotoh et al., 2010; Kodama et al., 2010; Kinch et al., 2022; Zou et al., 2023). However, roles of the other putative regulators are still unknown, and more studies should be performed to elucidate their regulatory actions on *V. parahaemolyticus* gene expression in future.

In conclusion, the biofilm formation, c-di-GMP production, swimming motility, zebrafish survival rate, cytoxicity and adherence activity of *V. parahaemolyticus* RIMD2210633 were significantly enhanced by Ca^2+^. However, Ca^2+^ had no effect on the growth, swarming motility, CPS production and hemolytic activity. A total of 459 genes were remarkably differentially expressed in response to Ca^2+^, including the key virulence genes, biofilm formation-associated genes and putative regulatory genes. DEGs involved in polar flagellum and T3SS1 were upregulated, whereas majority of those involved in regulatory functions and c-di-GMP metabolism were downregulated. The work helps us to understand how Ca^2+^ affects the behavior and gene expression of *V. parahaemolyticus* RIMD2210633. However, due to the limitation of sample collection for RNA-seq, the transcriptome data cannot reflect the dynamic response of *V. parahaemolyticus* RIMD2210633 to Ca^2+^ stimulation, and more works remain to be need to discover potential mechanisms.

## Data availability statement

The datasets presented in this study can be found in online repositories. The names of the repository/repositories and accession number(s) can be found in the article/Supplementary material.

## Ethics statement

Ethical approval was not required for the studies on animals in accordance with the local legislation and institutional requirements because only commercially available established cell lines were used.

## Author contributions

XL: Formal analysis, Investigation, Writing – original draft. JC: Investigation, Writing – original draft. MZ: Investigation, Writing – original draft. YinZ: Investigation, Writing – original draft. TZ: Investigation, Writing – original draft. YiqZ: Data curation, Formal analysis, Methodology, Supervision, Validation, Writing – review & editing. RL: Funding acquisition, Project administration, Resources, Supervision, Validation, Writing – review & editing.
